# Measuring anxiety in Lewy Body Disease – Which scale to choose?

**DOI:** 10.1016/j.prdoa.2021.100110

**Published:** 2021-09-28

**Authors:** Isabel Paniak, Simon J.G. Lewis, Kaylena A. Ehgoetz Martens

**Affiliations:** a Parkinson’s Disease Research Clinic, Brain and Mind Centre, University of Sydney, New South Wales, Australia; b Department of Kinesiology and Health Sciences, University of Waterloo, Ontario, Canada

## Abstract

**Background:**

Anxiety is among the most prevalent mood disorders in Lewy Body Disease (LBD) (i.e., Parkinson’s disease (PD), Dementia with Lewy bodies DLB), and those at-risk for developing LBD (e.g. isolated REM Sleep Behaviour Disorder (iRBD)). Yet, there is little consensus on which clinical scale best evaluates anxiety across synuclein-based diseases.

**Objective:**

This study compared the convergent validity of commonly used anxiety scales across PD, DLB and iRBD patients.

**Methods:**

Anxiety was assessed using the Hospital Anxiety and Depression Scale (HADS-A), State-Trait Anxiety Inventory (STAI), MDS-UPDRS Anxiety item, and the Parkinson Anxiety Scale (PAS) in 57 participants (17 PD, 16 DLB, and 23 iRBD).

**Results:**

Across all groups, PAS total score was significantly associated with trait anxiety (STAI-Y2), whilst HADS-A was associated with PAS total score in the PD and iRBD group. In DLB patients, HADS-A was weakly associated with PAS total score, and significantly correlated with PAS episodic anxiety. Notably, the anxiety item from the MDS-UPDRS did not correlate with any of the other anxiety outcome measures in any group.

**Conclusions:**

PAS and STAI-Y2 are the most suitable scales to assess anxiety in synuclein-based diseases. HADS-A showed strong convergent validity in PD and iRBD, it had weaker convergent validity in DLB. The UPDRS anxiety item did not correlate with any of the other anxiety measures, and thus may not be sensitive at detecting anxiety symptoms. Future work should validate anxiety scales in all Lewy Body Disease groups if they are to be implemented in prospective longitudinal cohorts.

## Highlights

• Parkinson Anxiety Scale is best suited to assess anxiety in synucleinopathies.
• Anxiety section of the HADS shows strong convergent validity in PD and iRBD.
• The UPDRS anxiety item did not correlate with any of the other anxiety scales.

## 1 Introduction

Anxiety is one of the most prevalent mood disorders in Lewy Body Disease, which comprises of both Parkinson’s disease (PD) and dementia with Lewy bodies (DLB) [1], [2], [3]. Upwards of 69% of the PD population experience symptoms of anxiety, and approximately one-third of PD patients satisfy the DSM criteria for a discrete anxiety disorder [4]. Likewise, anxiety is significantly more frequent in DLB (∼63%) compared to Alzheimer’s disease (27%) [5]. In both of these synucleinopathies (PD and DLB), anxiety often presents during the prodromal period (prior to clinical diagnosis). Given that isolated rapid eye movement (REM) sleep behaviour disorder (iRBD) is one of the best predictors of the development of these synucleinopathies, it may not be surprising that anxiety is also commonly reported in 15–23% of patients with iRBD [3], [6].

Given the impact of anxiety on quality of life, and the high prevalence of anxiety in those with LBD (i.e., PD, DLB), as well as those in at-risk populations (e.g., iRBD), it is important to evaluate these symptoms and their severity. However, it can be a challenge to select the appropriate rating scale given the variety of options available. Many of these scales have only been validated in PD rather than a spectrum of LBD including DLB or ‘prodromal LBD’. Therefore, the current aim of this short report was to evaluate the convergent validity of commonly used anxiety scales in a cohort of PD, DLB and iRBD patients to determine the overlap between the clinical tools that are commonly used to assess anxiety, and establish their suitability for quantifying anxiety across a spectrum of LBD patients in research and clinical settings.

## 2 Methods

Fifty-seven participants (23 iRBD, 17 PD, 17 DLB) were recruited from the Parkinson’s Disease Research Clinic at the Brian and Mind Centre, University of Sydney (see Table 1). All participants underwent a neurological assessment by a neurologist and movement disorder specialist (SJGL). Isolated RBD patients were confirmed using diagnostic polysomnography and did not satisfy diagnostic criteria for PD, DLB or MSA [7], [8]. Ethical approval was obtained from the University of Sydney Human Research and Ethics Committee and written informed consent was taken from each patient.

**Table 1:** Participant Demographics.

	iRBD (N = 23)	PD (N = 17)	DLB (N = 17)
Age (years)	66.7 ± 7.8	62.9 ± 10.4	73.1 ± 7.1
Male (%)	78%	65%	94%
Female (%)	22%	35%	6%
MoCA	27.7 ± 2.2	28.7 ± 1.3	18.3 ± 6.8
MDS-UPDRS III	7.6 ± 4.9	24.7 ± 10.8	36.3 ± 13.7
HADS-A	3.2 ± 2.5 (0–8)	4.5 ± 3.0 (0–11)	6.9 ± 4.7 (0–14)
UPDRS-I Anxiety	0.3 ± 0.47 (0–1)	0.41 ± 0.51 (0–1)	1.63 ± 1.2 (0–3)
PAS-Total	6.6 ± 5.2 (0–18)	9.4 ± 4.8 (3–17)	15.4 ± 5.4 (7–24)
PAS-Persistent	4.1 ± 3.1 (0–10)	6.2 ± 3.0 (1–12)	8.6 ± 3.6 (2–16)
PAS-Episodic	1.3 ± 1.4 (0–5)	1.5 ± 1.6 (0–6)	3.3 ± 2.6 (0–8)
PAS-Avoidant	1.2 ± 1.9 (0–6)	1.7 ± 1.7 (0–5)	3.6 ± 2.3 (0–7)
STAI-Trait	31.9 ± 9.5 (21–60)	36.1 ± 8.6 (23–52)	42.1 ± 9.3 (24–57)
STAI-State	27 ± 6.5 (20–44)	29 ± 7.3 (20–46)	37.3 ± 9.5 (22–58)

Values include Mean ± Standard Deviation (Min-Max)

Anxiety symptoms were assessed using the Hospital Anxiety and Depression Scale anxiety subscale (HADS-A), the State-Trait Anxiety Inventory (STAI), the Parkinson’s Anxiety Scale (PAS), and the anxiety item from the Movement Disorder Society Unified Parkinson’s Disease Rating Scale (UPDRS) section I. Spearman’s rank correlations were used to examine the convergent validity amongst the anxiety rating scales across the different patient groups.

## 3 Results

Across all groups, the total score on the PAS was significantly correlated with trait anxiety (measured by the STAI-Y2 form) (iRBD: r = 0.80, p < 0.001; PD: r = 0.68, p = 0.003; DLB: r = 0.58, p = 0.018). Trait anxiety was also correlated with the persistent anxiety sub score on the PAS (iRBD: r = 0.73, p < 0.001; PD: r = 0.58, p = 0.015; DLB: r = 0.61, p = 0.012) and the episodic anxiety sub score on the PAS in the iRBD cohort (r = 0.53, p = 0.011).

Interestingly, HADS-A total was correlated with the PAS-total in iRBD (r = 0.73, p < 0.001), PD (r = 0.58, p = 0.016), but not in DLB (r = 0.47, p = 0.09). More specifically, HADS-A total was also correlated with persistent anxiety PAS sub score in the iRBD and PD groups (iRBD: r = 0.66, p = 0.001; PD: r = 0.54, p = 0.024) and with the episodic anxiety PAS sub score in all groups (iRBD: r = 0.65, p = 0.001; PD: r = 0.49, p = 0.044; DLB: r = 0.55, p = 0.043). HADS-A total was also correlated with Trait anxiety in the iRBD (r = 0.67, p = 0.001) and both trait and state anxiety in the PD cohort (trait: r = 0.67, p = 0.003; state: r = 0.6, p = 0.015). The avoidant PAS sub score was not related to any other anxiety scale measures. This was consistent across all groups. There were also no correlations between ratings on the UPDRS anxiety item, and the PAS score (or sub scores), nor HADS-A total across all patient groups.

In sum, convergent validity across anxiety was predominantly seen in the PD and iRBD cohort, and less evident when using these instruments in DLB cohorts. Notably, the rating on the anxiety item from the UPDRS-I was not correlated to either the HADS-A or PAS in any patient groups.

## 4 Discussion

Given that anxiety is a common mood disturbance in PD, DLB and iRBD, and remains one of the top unmet needs in the PD community [9], it is critical to assess anxiety symptoms. Yet, there is a dearth of research on anxiety within these groups, including an understanding of which scales might be most appropriate. Here, we examined the convergent validity across common anxiety scales separately in PD, DLB and iRBD cohorts. Our study revealed most notably that the UPDRS anxiety item was not related to any of the other anxiety scales and may be the culprit for why anxiety remains underdiagnosed and undertreated in these populations. One possible explanation for this lack of correlation could be that the item 1.4 in the MDS-UPDRS was the only scale that was completed by an examiner which may have led patients to under-report the severity of their anxiety symptoms, or how it impacted their daily life compared to when questionnaires were self-administered. Another consideration is that the range of the UPDRS item was much less than other anxiety scales and also may have reduced the ability to detect a meaningful correlation. In contrast, the PAS-total was consistently related to trait anxiety across all of the different clinical groups and may be the best clinical tool for assessing anxiety across the spectrum of LBD.

The greatest convergence across the majority of scales (PAS, HADS-A, STAI) was found when employed in the PD and iRBD cohort, whereas little convergence was found in DLB. For example, in both the iRBD and PD cohort, HADS-A was correlated with STAI-trait anxiety, PAS-total, as well as PAS-persistent and PAS-episodic. This suggests that in PD and iRBD, HADS-A has high convergent validity, as does PAS given it was also associated with scores on the STAI-trait and HADS-A. In contrast, the only correlation between scales that was found for the DLB cohort was between PAS-total and STAI-trait anxiety, highlighting that HADS-A may not be as useful for assessing anxiety in DLB. The HADS is a screening instrument, rather than a diagnostic tool [10]. Previous research has noted that the psychometric properties of the HADS are satisfactory [10], although it has been found to have good interrater and test–retest reliability for detecting anxiety in patients with PD [11]. However, in the current study HADS-A showed weaker-moderate correlations to PAS-total (r = 0.47) and trait anxiety (r = 0.22) when administered to DLB patients, calling into question its clinical utility. The absence of correlations within DLB suggests that anxiety may present differently within this group which may not be best captured with HADS-A. It is noteworthy that anxiety ratings were higher and more variable in DLB patients compared to the other cohorts. It is possible that fatigue or fluctuations in attention may have impacted the consistency in reporting anxiety symptoms across assessments. To this end, a caveat of this study is the small sample sizes of each group which limits the generalizability of this work. Future work should consider performing a sample size estimation, in order to fully address whether this clinical tool has satisfactory psychometric properties work when carried out in lager samples, particularly when employed in DLB, and further clarify anxiety symptomology in DLB.

The PAS was developed specifically to overcome the shortcomings of other anxiety scales that were used in PD [12]. The advantages of the PAS include its clinometric properties, internal consistency, test–retest reliability, inter-rater reliability, sensitivity and specificity [12]. However, the downsides of anxiety scales, including the PAS, are their inability to detect unique presentations of anxiety [12]. Although the PAS was developed as an anxiety scale for PD, it has only been validated for PD without dementia, and to our knowledge has not been investigated in DLB or iRBD cohorts. Therefore, it was unclear prior to this study whether it would be useful in detecting anxiety in DLB or iRBD. Our findings showed that in all three groups, STAI-trait was correlated with both PAS-persistent and PAS-total, suggesting it captures more general and long-standing anxiety symptoms. Within iRBD and PD, PAS- episodic was also correlated with both HADS-A and STAI-trait, further evidencing the convergence validity. These findings suggest that the PAS is able to detect anxiety in LBD groups with and without dementia.

While this study found overlap amongst a small set of anxiety scales in PD, DLB and iRBD, there are a number of other clinical scales that could be considered in future research such as the Beck Anxiety Inventory, Hamilton Anxiety Scale and the Generalized Anxiety Scale. Future work is needed to elucidate which scale most accurately captures anxiety symptomology across synuclein-based disorders. Additionally, this study was limited to anxiety symptoms captured in a cross-sectional clinical setting, which is not always representative of the symptoms experienced in more natural settings, such as at home. Future studies might consider at-home monitoring of anxiety using mobile apps for tracking physiology (e.g. heart rate variability), symptom severity and daily fluctuations as their disease progresses. Although anxiety has been associated with accelerated ageing and brain degeneration, it remains unclear how anxiety might impact disease progression across LBD.

In conclusion, anxiety is prevalent, yet often remains untreated within PD, DLB and iRBD. Based on our findings, PAS and STAI are the most suitable scales to be used to assess anxiety in PD, iRBD and DLB, whilst the HADS-A shows strong convergent validity in PD and iRBD, it had weaker convergent validity to the other anxiety scales in DLB. In our sample, the UPDRS anxiety item did not correlate with any of the other anxiety measures, and thus may not be sensitive at detecting anxiety symptoms, however further research is needed to determine the generalisability of these results. Given that scales remain our best tool to accurately screen for anxiety and quantify symptom severity, as well as to evaluate the effectiveness of therapeutic treatment, further research is needed to establish gold standard clinical assessments for anxiety symptoms in DLB and iRBD.

## CRediT authorship contribution statement

**Isabel Paniak:** Data curation, Writing – original draft. **Simon J.G. Lewis:** Writing – review & editing. **Kaylena A. Ehgoetz Martens:** Conceptualization, Writing – review & editing, Supervision.

## Declaration of Competing Interest

The authors declare that they have no known competing financial interests or personal relationships that could have appeared to influence the work reported in this paper.

## References

[1] Coakeley S., Martens K.E., Almeida Q.J. (2014). Management of anxiety and motor symptoms in Parkinson’s disease. Expert Rev. Neurother..

[2] Chaudhuri K.R., Healy D.G., Schapira A.HV. (2006). National Institute for Clinical Excellence, Non-motor symptoms of Parkinson's disease: diagnosis and management. Lancet Neurol..

[3] Chiu N.K.H., Ehgoetz Martens K.A., Mok V.C.T., Lewis S.J.G., Matar E. (2021). Prevalence and predictors of mood disturbances in idiopathic REM sleep behaviour disorder. J. Sleep Res..

[4] Broen M.P.G., Narayen N.E., Kuijf M.L., Dissanayaka N.N.W., Leentjens A.F.G. (2016). Prevalence of anxiety in Parkinson's disease: a systematic review and meta-analysis. Mov. Disord..

[5] Segers K., Benoit F., Meyts J.M., Surquin M. (2019). Anxiety symptoms are quantitatively and qualitatively different in dementia with Lewy bodies than in Alzheimer’s disease in the years preceding clinical diagnosis. Psychogeriatrics.

[6] Postuma R.B., Iranzo A., Hu M. (2019). Risks and predictors of dementia and Parkinsonism in idiopathic REM sleep behaviour disorder: a multicentre study. Brain.

[7] McKeith I., Boeve B., Dickson D.W. (2017). Diagnosis and management of dementia with Lewy bodies: fourth consensus report of the DLB Consortium. Neurology.

[8] Gilman S., Wenning G.K., Low P.A. (2008). Consensus statement on the diagnosis of multiple system atrophy. Neurology.

[9] Pontone G.M., Dissanayaka N., Apostolova L. (2019). Report from a multidisciplinary meeting on anxiety as a non-motor manifestation of Parkinson’s disease. NPJ Parkinsons Dis..

[10] Marinus J., Leentjens A.F.G., Visser M., Stiggelbout A.M., van Hilten J.J. (2002). Evaluation of the Hospital Anxiety and Depression Scale in patients with Parkinson’s Disease. Clin. Neuropharmacol..

[11] Leentjens A.F.G., Dujardin K., Marsh L., Richard I.H., Starkstein S.E., Martinez-Martin P. (2011). Anxiety rating scales in Parkinson's disease: a validation study of the Hamilton anxiety rating scale, the Beck anxiety inventory, and the hospital anxiety and depression scale. Mov. Disord..

[12] Leentjens A.F.G., Dujardin K., Pontone G.M., Starkstein S.E., Weintraub D., Martinez-Martin P. (2014). The Parkinson Anxiety Scale (PAS): development and validation of a new anxiety scale. Mov. Disord..

